# Amikacin activity against carbapenem-resistant *Klebsiella pneumoniae* without phenotypic aminoglycoside resistance: impact of aminoglycoside-modifying enzyme (AME) gene *aac(6′)-Ib-cr in vitro* and *in vivo*

**DOI:** 10.1093/jacamr/dlag239

**Published:** 2026-11-02

**Authors:** Jaehoon Kim, Jae Eun Seong, Youn-Jung Lee, Sora Park, Min Han, Yongseop Lee, Jung Ah Lee, Jung Ho Kim, Jin Young Ahn, Su Jin Jeong, Nam Su Ku, Joon-Sup Yeom, Hyukmin Lee, Jun Yong Choi

**Affiliations:** Division of Infectious Diseases, Department of Internal Medicine, Yonsei University College of Medicine, 50-1 Yonsei-ro, Seodaemun-gu, Seoul 03722, Republic of Korea; Division of Infectious Diseases, Department of Internal Medicine, Seoul Medical Center, 156, Sinnae-ro, Jungnang-gu, Seoul 02053, Republic of Korea; Division of Infectious Diseases, Department of Internal Medicine, Yonsei University College of Medicine, 50-1 Yonsei-ro, Seodaemun-gu, Seoul 03722, Republic of Korea; Division of Infectious Diseases, Department of Internal Medicine, Yonsei University College of Medicine, 50-1 Yonsei-ro, Seodaemun-gu, Seoul 03722, Republic of Korea; Division of Infectious Diseases, Department of Internal Medicine, Yonsei University College of Medicine, 50-1 Yonsei-ro, Seodaemun-gu, Seoul 03722, Republic of Korea; Division of Infectious Diseases, Department of Internal Medicine, Yonsei University College of Medicine, 50-1 Yonsei-ro, Seodaemun-gu, Seoul 03722, Republic of Korea; Division of Infectious Diseases, Department of Internal Medicine, Yonsei University College of Medicine, 50-1 Yonsei-ro, Seodaemun-gu, Seoul 03722, Republic of Korea; Division of Infectious Diseases, Department of Internal Medicine, Yonsei University College of Medicine, 50-1 Yonsei-ro, Seodaemun-gu, Seoul 03722, Republic of Korea; Division of Infectious Diseases, Department of Internal Medicine, Yonsei University College of Medicine, 50-1 Yonsei-ro, Seodaemun-gu, Seoul 03722, Republic of Korea; Division of Infectious Diseases, Department of Internal Medicine, Yonsei University College of Medicine, 50-1 Yonsei-ro, Seodaemun-gu, Seoul 03722, Republic of Korea; Division of Infectious Diseases, Department of Internal Medicine, Yonsei University College of Medicine, 50-1 Yonsei-ro, Seodaemun-gu, Seoul 03722, Republic of Korea; Division of Infectious Diseases, Department of Internal Medicine, Yonsei University College of Medicine, 50-1 Yonsei-ro, Seodaemun-gu, Seoul 03722, Republic of Korea; Department of Laboratory Medicine, Yonsei University College of Medicine, 50-1 Yonsei-ro, Seodaemun-gu, Seoul 03722, Republic of Korea; Division of Infectious Diseases, Department of Internal Medicine, Yonsei University College of Medicine, 50-1 Yonsei-ro, Seodaemun-gu, Seoul 03722, Republic of Korea

## Abstract

**Objectives:**

Aminoglycosides are often combined with β-lactams for the treatment of carbapenem-resistant Enterobacterales, but the impact of aminoglycoside-modifying enzyme (AME) genes on treatment efficacy in phenotypically susceptible isolates remains unclear. We evaluated whether *aac(6′)-Ib-cr*, the AME gene with recognized activity against amikacin, attenuates amikacin efficacy in KPC-producing *Klebsiella pneumoniae*.

**Methods:**

Thirteen clinical isolates of KPC-producing *K. pneumoniae* without phenotypic aminoglycoside resistance (amikacin MIC ≤4 mg/L; median 2, range 0.5–3 mg/L) were studied. Three were AME-negative; 10 were AME-positive, carrying *aac(6′)-Ib-cr*, *aac(3)-IIa* and *strA/strB*. Amikacin activity was assessed by time-kill assays and a *Galleria mellonella* survival model, compared with ceftazidime–avibactam.

**Results:**

In time-kill assays, amikacin killing at 24 h was greater in AME-negative isolates than in AME-positive isolates (mean 4.28 ± 1.30 versus 2.41 ± 2.16 log_10_ cfu/mL), with more regrowth by 72 h in AME-positive isolates (median 1.94 [0–5.82] versus 0 [0–3.32] log_10_). In the survival model, amikacin reduced larval mortality versus saline (HR, 0.32; 95% CI, 0.24–0.43; *P* < 0.001). The interaction between AME-positive status and amikacin (HR, 2.11; 95% CI, 1.54–2.90; *P* < 0.001) indicated reduced efficacy that persisted after adjustment for amikacin MIC (adjusted HR, 2.09; 95% CI, 1.52–2.86; *P* < 0.001). Ceftazidime–avibactam reduced mortality (HR, 0.24; 95% CI, 0.18–0.33; *P* < 0.001) with no significant interaction with AME gene status (HR, 1.03; 95% CI, 0.71–1.48; *P* = 0.894).

**Conclusion:**

Carriage of *aac(6′)-Ib-cr* was associated with reduced amikacin efficacy despite phenotypic susceptibility, independently of amikacin MIC, consistent with a treatment effect modifier rather than a binary resistance determinant. Genotyping of *aac(6′)-Ib-cr* may help guide aminoglycoside use in carbapenem-resistant *Klebsiella pneumoniae* infections.

## Introduction

Carbapenem-resistant Enterobacterales (CREs) represent an escalating threat to global health, as the infections they induce are associated with limited therapeutic options and high mortality rates.^1^ Among CREs, carbapenem-resistant *Klebsiella pneumoniae* (CRKP) infection outbreaks are predominantly caused by the dissemination of carbapenemase-producing strains, particularly those harbouring *bla*_KPC_.^2,3^ The introduction of ceftazidime–avibactam, a β-lactam/β-lactamase inhibitor combination targeting Ambler class A β-lactamases, significantly improved clinical outcomes in infections caused by KPC–producing *K. pneumonia*.^4^ However, resistance to ceftazidime–avibactam has been increasingly reported, necessitating continued reliance on legacy agents, such as aminoglycosides, in clinical practice, frequently as part of combination regimens.^5,6^

Appropriate antimicrobial therapy is critical for improving the outcomes of infectious diseases, including severe sepsis, along with timely source control and appropriate management of organ dysfunction.^7,8^ AST, which is based on MICs, is the cornerstone of antibiotic selection. However, the degree of discordance between phenotypic susceptibility and clinical outcomes is increasingly recognized, particularly in multidrug-resistant Gram-negative infections.^9^ Aminoglycoside-modifying enzymes (AMEs) differ in substrate specificity: AAC(3) and the APH(3″)-Ib/APH(6)-Id phosphotransferases (encoded by *strA/strB*) do not act on amikacin, whereas AAC(6′)-Ib is the principal enzyme acting on it.^10–12^ AAC(6′)-Ib–producing CRKP may regrow at clinically achievable amikacin concentrations despite susceptible MICs.^13,14^

*Galleria mellonella* larval infection models are widely used to test bacterial virulence and antimicrobial efficacy.^15^ This model allows incubation at 37°C and recapitulates key aspects of mammalian innate immunity, including both humoral and cellular responses.^16–18^ They also offer practical advantages, including being low cost, ease of handling, and being exempt from the ethical constraints associated with mammalian models.^19^

Evidence directly linking *aac(6′)-Ib* carriage to the efficacy of aminoglycoside treatment, particularly in isolates that remain phenotypically susceptible, is limited. In this study, we investigated the activity of amikacin against KPC-producing *K. pneumoniae* isolates without phenotypic aminoglycoside resistance, stratified by AME gene *aac(6′)-Ib-cr* status, using *in vitro* time-kill assays and an *in vivo G. mellonella* survival model. Furthermore, we compared these findings with the activity of ceftazidime–avibactam to delineate AME-specific effects on antimicrobial efficacy.

## Methods

### Bacterial isolates and whole-genome sequencing

Thirteen carbapenem-resistant KPC-producing *K. pneumoniae* isolates were obtained from patients with bacteraemia at the Severance Hospital, Seoul, Republic of Korea between September 2022 and December 2023. The reference strain (NCCP 14764) was obtained from the Korea National Culture Collection of Pathogens. All isolates were stored at −80°C in cation-adjusted Mueller–Hinton broth supplemented with 20% glycerol and were sub-cultured on blood agar base plates containing 5% defibrinated sheep blood before each experiment. Amikacin was purchased from Sigma-Aldrich (St. Louis, MO). Ceftazidime–avibactam (4:1) was purchased from Pfizer (New York, NY, USA). Stock solutions of each antimicrobial agent were freshly prepared weekly, aliquoted for single use, and stored at −20°C until use.^20^ Whole-genome sequencing was performed on all isolates. Genomic DNA was extracted using the Chemagic DNA Blood200 kit (Invitrogen, Waltham, MA, USA). Sequencing libraries were prepared using the Library Preparation Enzymatic Fragmentation Kit 2.0 (Twist Bioscience, San Francisco, CA, USA) with 100 ng of genomic DNA. Paired-end sequencing was conducted using an Illumina NovaSeq 6000 Dx platform (Illumina, San Diego, CA, USA). Genome assemblies were analysed using the Kleborate software (version 3.2.4). Acquired AME genes were identified using the *AGly_acquired* output with the default settings. The sequence type and virulence loci were derived from Kleborate.

### Time-kill assays

Time-kill assays were conducted to evaluate bacterial killing and regrowth following exposure to amikacin and ceftazidime–avibactam against *K. pneumoniae* isolates. A single colony from an overnight culture was inoculated into cation-adjusted Mueller–Hinton broth and incubated at 37°C with shaking to reach the exponential growth phase. The bacterial suspension was adjusted to a turbidity equivalent to a McFarland standard of 0.5, and subsequently diluted to achieve an initial inoculum of approximately 1.5 × 10^5^ cfu/mL, which was confirmed by viable colony counts.

MICs were determined using Etest strips (BioMèrieux) according to the manufacturer's instructions. Each isolate was exposed to amikacin or ceftazidime–avibactam at 1 × MIC under static concentration conditions (no supplement dosing), and drug-free growth controls were included. Samples were collected at 0, 2, 6, 24, 48, and 72 h, serially diluted in 0.9% sodium chloride, and plated onto Mueller–Hinton agar for colony enumeration following incubation at 37°C. Bacterial killing was assessed by plotting log_10_ cfu/mL over the 72 h observation period. Bactericidal activity was defined as a  ≥ 3 log_10_ cfu/mL reduction in bacterial density compared with the starting inoculum. All experiments were performed once for each isolate.

### Galleria mellonella survival model

Final-instar *G. mellonella* larvae were obtained from a commercial supplier (S Worm Company, Korea) and were used for experiments within 24 h of arrival. Healthy larvae without grey discoloration and weighing 150–200 mg were randomly selected for the experiment. For each experimental condition, 20 larvae were grouped to ensure a uniform total weight. A 10 μL inoculum of *K. pneumoniae* was injected into the last left proleg using an insulin syringe. The bacterial inoculum for each isolate was predetermined based on preliminary experiments to achieve >50% mortality within 72 h and ranged from 1.5 × 10^7^ to 1.5 × 10^8^ cfu/mL. One hour after bacterial infection, amikacin, ceftazidime-avibactam, or PBS was administered as a 10 μL injection into the last right proleg. For each experiment, a positive control (*K. pneumoniae* NCCP 14764) and a negative control (larvae injected with saline alone, without bacterial challenge) were included to evaluate the differences between the clinical and reference strains and to monitor background mortality. Doses were selected on the basis of preliminary dose-finding experiments in all isolates, in which survival increased in a dose-dependent manner for both agents. Both agents were administered at 32 mg/L (0.32 µg per larva), a concentration that exceeded the MIC of all isolates and conferred a clear survival benefit over untreated controls. After injection, larvae were incubated at 37°C, and survival was recorded at 6, 24, 48, and 72 h. Each experiment was performed in triplicate, comprising 60 larvae per condition in total.

### Statistical analysis

The survival data of the *G. mellonella* models were analysed using the Kaplan–Meier method. Differences in survival between the treatment groups were initially assessed using the log-rank (Mantel–Cox) test. To evaluate the independent effects of antimicrobial treatment and the modifying effect of AME gene status on survival, Cox proportional hazard regression models were constructed, including interaction terms between AME status and treatment. To examine whether the reduced amikacin efficacy in AME-positive isolates was independent of amikacin MIC, a sensitivity analysis was performed by adding the log_2_-transformed amikacin MIC to the model. A further model incorporating an MIC × treatment interaction was fitted to assess whether MIC specifically modified the amikacin treatment effect. All statistical analyses were performed using R software (version 4.5.2).

This study was approved by the Institutional Review Board of Severance Hospital (IRB No. 4-2024-1543) and the requirement for informed consent was waived because of the use of anonymised bacterial isolates.

## Results

The MIC values obtained using the Etest are listed in Table 1. All AME-negative (S1–S3) and AME-positive isolates (R1–R10) were categorized as susceptible to amikacin (MIC ≤4 mg/L) and ceftazidime–avibactam (MIC ≤ 8/4 mg/L), according to the CLSI breakpoints (M100). The whole-genome sequencing results are presented in Table 2. All the isolates carried the *bla*_KPC-2_ carbapenemase gene. All but S3 belonged to ST307, and S3 belonged to ST65. No AME genes were detected in S1–S3, whereas all R1–R10 isolates harboured *aac(6′)-Ib-cr, aac(3)-IIa* and *strA/strB*.

**Table 1.** MICs of *K. pneumoniae* isolates, determined using the Etest

Strain	Amikacin		Ceftazidime–avibactam	
	MIC (mg/L)	Interpretive category^a^	MIC (mg/L)	Interpretive category^b^
Positive control^c^	1	S	0.125	S
S1	0.5	S	0.5	S
S2	1	S	2	S
S3	1	S	0.38	S
R1	2	S	1	S
R2	2	S	1.5	S
R3	3	S	1.5	S
R4	3	S	0.38	S
R5	2	S	0.5	S
R6	3	S	1	S
R7	3	S	1.5	S
R8	1.5	S	1.5	S
R9	2	S	4	S
R10	2	S	1	S

S, susceptible; I, intermediate; R, resistant.

^a^Susceptibility breakpoints for amikacin: susceptible, ≤4 mg/L; resistant, ≥16 mg/L, according to CLSI M100 (35th edition, 2025).

^b^Susceptibility breakpoints for ceftazidime–avibactam; susceptible, ≤8/4 mg/L; resistant, ≥16/4 mg/L, according to CLSI M100 (35th edition, 2025).

^c^Positive control refers to *K. pneumoniae* NCCP 14764 included for reference comparison.

**Table 2.** Whole-genome sequencing characteristics of the *K. pneumoniae* isolates

Strain	ST	Carbapenemase	ESBL gene^a^	AME genes	Virulence genes	K-locus	O-type
S1	ST307	KPC-2	—	—	*ybt16 (ICEKp12)*	KL102	O2β
S2	ST307	KPC-2	CTX-M-15	—	*—*	KL102	O2β
S3	ST65	KPC-2	—	—	*iuc1, iro1, rmpA2*	K2	O1αβ, O2β
R1	ST307	KPC-2	CTX-M-15	*aac(6′)-Ib-cr; aac(3)-IIa; strA/strB*	*ybt16 (ICEKp12)*	KL102	O2β
R2	ST307	KPC-2	CTX-M-15	*aac(6′)-Ib-cr; aac(3)-IIa; strA/strB*	*ybt16 (ICEKp12)*	KL102	O2β
R3	ST307	KPC-2	CTX-M-15	*aac(6′)-Ib-cr; aac(3)-IIa; strA/strB*	*ybt16 (ICEKp12)*	KL102	O2β
R4	ST307	KPC-2	CTX-M-15	*aac(6′)-Ib-cr; aac(3)-IIa; strA/strB*	*ybt16 (ICEKp12)*	KL102	O2β
R5	ST307	KPC-2	CTX-M-15	*aac(6′)-Ib-cr; aac(3)-IIa; strA/strB*	*ybt16 (ICEKp12)*	KL-null	O2β
R6	ST307	KPC-2	CTX-M-15	*aac(6′)-Ib-cr; aac(3)-IIa; strA/strB*	*ybt16 (ICEKp12)*	KL-null	O2β
R7	ST307	KPC-2	CTX-M-15	*aac(6′)-Ib-cr; aac(3)-IIa; strA/strB*	*ybt16 (ICEKp12)*	KL102	O2β
R8	ST307	KPC-2	CTX-M-15	*aac(6′)-Ib-cr; aac(3)-IIa; strA/strB*	*ybt16 (ICEKp12)*	KL102	O2β
R9	ST307	KPC-2	CTX-M-15	*aac(6′)-Ib-cr; aac(3)-IIa; strA/strB*	*ybt16 (ICEKp12)*	KL102	O2β
R10	ST307	KPC-2	CTX-M-15	*aac(6′)-Ib-cr; aac(3)-IIa; strA/strB*	—	KL102	O2β

ST, sequence type; ESBL, extended-spectrum β-lactamase; AME, aminoglycoside-modifying enzyme.

^a^ESBL positivity defined by the presence of *bla*_CTX-M-15_.

The results of the time-kill assays are shown in Figure 1. Detailed 24 h killing data and regrowth are provided in Table S1 (available as Supplementary data at *JAC-AMR* Online). Amikacin demonstrated bactericidal activity (≥3 log_10_ cfu/mL reduction) at 24 h in two of three AME-negative isolates (S2 and S3), which was sustained through 72 h, whereas S1 showed only a 2.8 log_10_ reduction followed by regrowth. Among AME-positive isolates, responses were heterogeneous: six isolates (R2, R3, R6, R7, R9, and R10) reached the bactericidal threshold at 24 h, but killing was frequently not sustained, with regrowth by 48–72 h in most isolates, whereas R4 and R8 showed net growth throughout. Overall, sustained killing over 72 h was more frequent in AME-negative isolates, which showed less regrowth from nadir to 72 h than AME-positive isolates (median 0 [0–3.32] versus 1.94 [0–5.82] log_10_). Ceftazidime–avibactam showed rapid killing against most isolates, with variable activity at later time points in several AME-positive isolates.

**Figure 1.**
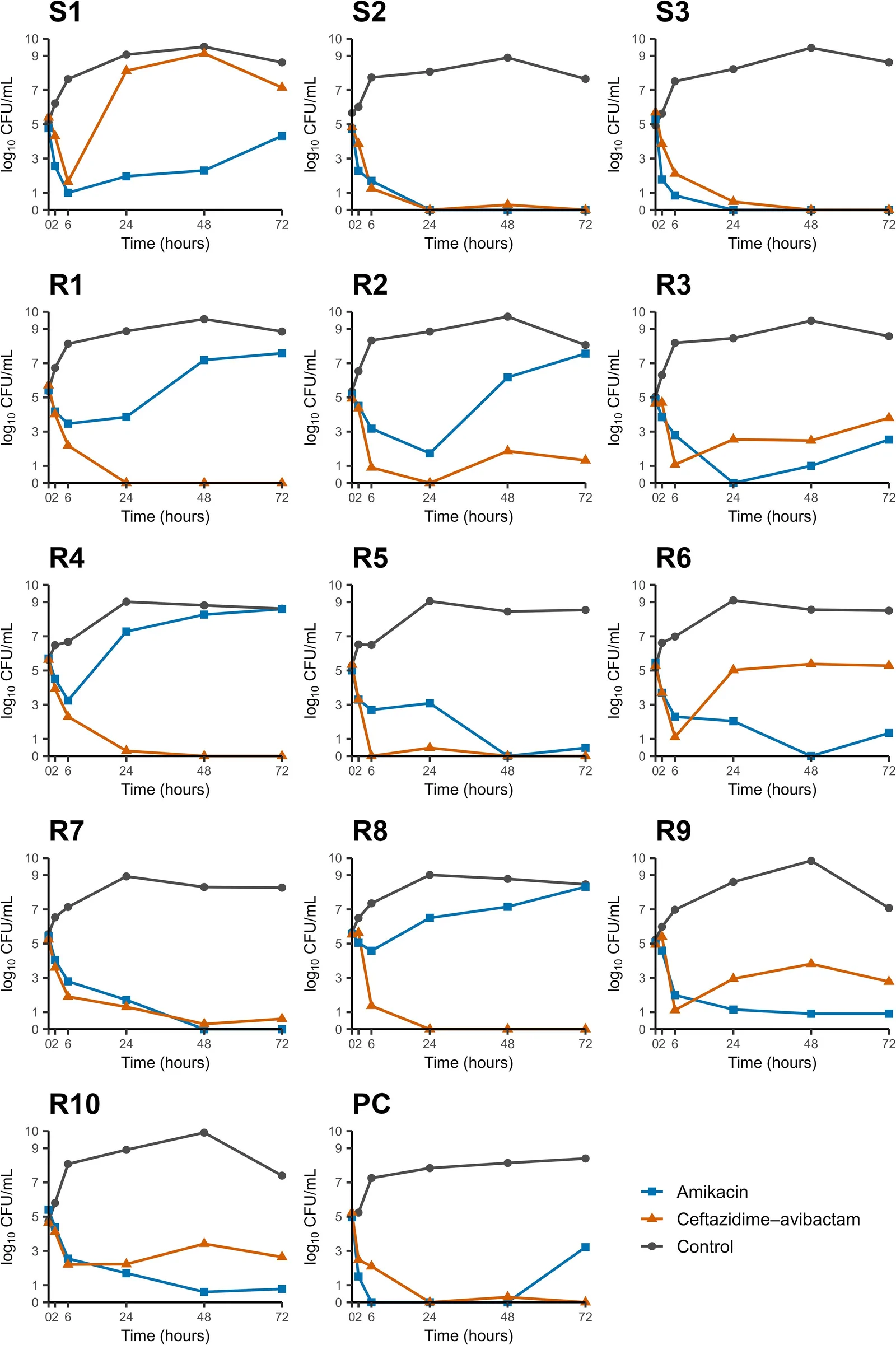
Time–kill curves of amikacin and ceftazidime–avibactam against KPC-producing *K. pneumoniae* isolates. S1−S3 denote AME-negative isolates, R1−R10 denote AME-positive isolates, and PC denotes the positive control strain. Amikacin and ceftazidime–avibactam were tested at 1× MIC (determined by the Etest). Viable cell counts were measured at 0, 2, 6, 24, 48, and 72 h. Bactericidal activity was defined as ≥3 log10 cfu/mL reduction in bacterial density from the initial inoculum. Each panel represents an isolate.

The survival outcomes in the *G. mellonella* infection model are shown in Figure 2. Amikacin treatment significantly improved the survival of larvae infected with the AME-negative isolates compared with that of those infected with AME-positive isolates. In contrast, ceftazidime–avibactam treatment was associated with improved survival regardless of the AME gene status. Survival curves for the positive control and the negative control are shown in Figure S1; background mortality in the negative control was minimal.

**Figure 2.**
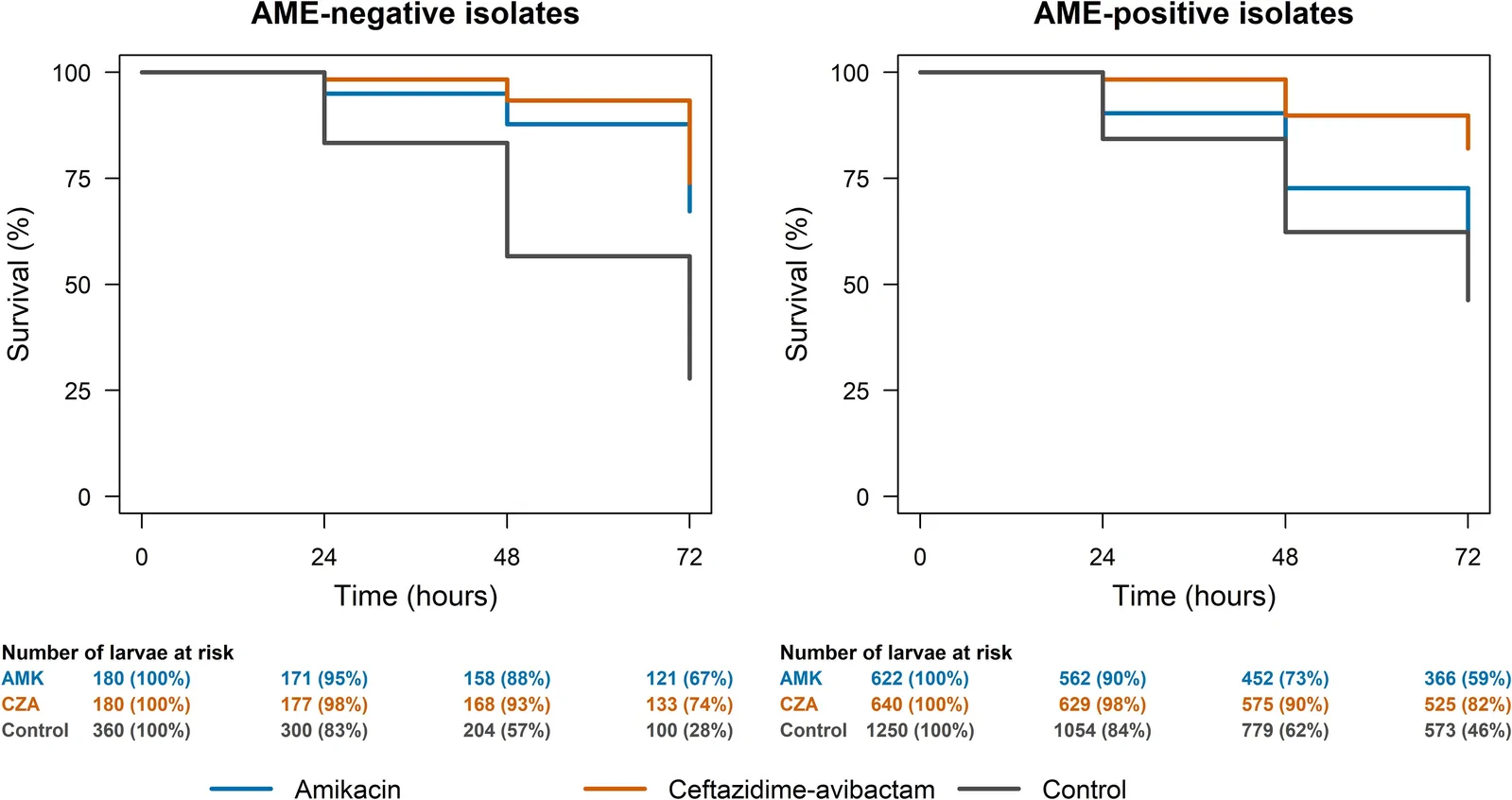
Kaplan–Meier survival curves of *G. mellonella* larvae infected with KPC-producing *K. pneumoniae* isolates according to AME status. The larvae were infected with AME-negative isolates (S1−S3), AME-positive isolates (R1−R10). One hour after infection, the larvae were administered amikacin, ceftazidime–avibactam, or a saline control. The antibiotics were administered at a concentration of 32 mg/L. Each condition included 20 larvae per experiment, and the experiments were performed in triplicate. Cell survival was monitored for 72 h. AMK, Amikacin; CZA, Ceftazidime–avibactam.

The results of the Cox proportional hazards regression analysis are shown in Table 3. Under saline treatment, larvae infected with AME-positive isolates exhibited a lower risk of death than those infected with AME-negative isolates (HR, 0.67; 95% CI, 0.58–0.77; *P* < 0.001). Amikacin treatment was associated with a reduced risk of death compared with saline (HR, 0.32; 95% CI, 0.24–0.43; *P* < 0.001), as was ceftazidime–avibactam versus saline (HR, 0.24; 95% CI, 0.18–0.33; *P* < 0.001). An interaction term indicates whether the effect of amikacin treatment differed according to another factor (e.g. AME status, MIC). The interaction between AME-positive status and amikacin treatment yielded a hazard ratio of 2.11 (95% CI, 1.54–2.90; *P* < 0.001), indicating a reduced amikacin treatment effect in AME-positive isolates. No significant interaction was observed between AME status and ceftazidime–avibactam treatment (HR, 1.03; 95% CI, 0.71–1.48; *P* = 0.894). After adjustment for amikacin MIC, the AME-positive × amikacin interaction remained significant (adjusted HR, 2.09; 95% CI, 1.52–2.86; *P* < 0.001), and higher amikacin MIC was independently associated with increased mortality (HR per doubling dilution, 1.47; 95% CI, 1.28–1.69; *P* < 0.001). When an MIC × treatment interaction was included, the MIC × amikacin term was not significant (HR, 0.97; 95% CI, 0.68–1.37; *P* = 0.85), whereas the AME-positive × amikacin interaction persisted (HR, 2.19; 95% CI, 1.20–4.01; *P* = 0.011).

**Table 3.** Cox proportional hazards regression analysis of the survival of infection model *G. mellonella*

Variable	Unadjusted HR^a^ (95% CI)	*P*-value	MIC-adjusted HR^a^ (95% CI)	*P*-value
AME status				
AME-positive versus AME-negative	0.67 (0.58–0.77)	<0.001	0.38 (0.29–0.49)	<0.001
Treatment				
Amikacin versus Saline	0.32 (0.24–0.43)	<0.001	0.32 (0.24–0.43)	<0.001
Ceftazidime–avibactam versus saline	0.24 (0.18–0.33)	<0.001	0.24 (0.18–0.33)	<0.001
Amikacin MIC				
Per log_2_ dilution increase	—	—	1.47 (1.28–1.69)	<0.001
Interaction terms				
AME-positive × Amikacin	2.11 (1.54–2.90)	<0.001	2.09 (1.52–2.86)	<0.001
AME-positive × Ceftazidime–avibactam	1.03 (0.71–1.48)	0.894	1.02 (0.70–1.47)	0.936

AME, aminoglycoside-modifying enzyme.

^a^The unadjusted model included main effects for AME status and treatment and their interaction terms. The MIC-adjusted model additionally included log_2_-transformed amikacin MIC as a covariate.

AME-negative isolates and saline served as reference groups.

## Discussion

In this study, amikacin activity against phenotypically susceptible CRKP was associated with AME gene *aac(6′)-Ib-cr* carriage. *In vitro*, sustained killing over 72 h was more frequent in AME-negative isolates, although individual responses overlapped between groups. In the *G. mellonella* model, the interaction between AME status and amikacin treatment was associated with reduced efficacy in AME-positive isolates (HR, 2.11; 95% CI, 1.54–2.90), an association that persisted after adjustment for amikacin MIC (adjusted HR, 2.09; 95% CI, 1.52–2.86). The MIC × amikacin interaction was not significant whereas the AME-positive × amikacin interaction persisted after MIC adjustment; thus, *aac(6*′*)-Ib-cr*, rather than MIC alone, appears to have acted as the treatment effect modifier.

These findings extend previous observations on *aac(6*′*)-Ib* and amikacin activity. Butler *et al.*^14^ reported progressively reduced aminoglycoside killing with repeated dosing against KPC-producing *K. pneumoniae* harbouring *aac(6*′*)-Ib*, although MIC remained a reasonable predictor of amikacin activity in their model. Bremmer *et al.*^13^ observed regrowth at clinically achievable amikacin concentrations in AAC(6′)-Ib–producing CRKP despite MICs within the susceptible-to-intermediate range. The regrowth we observed in AME-positive isolates is consistent with these reports, probably reflecting progressive AAC(6′)-Ib-cr–mediated inactivation of amikacin that would reduce effective drug exposure over time under single-dose conditions, although drug concentrations were not measured. In addition, a Korean cohort study found no clinical benefit of aminoglycoside therapy in CRKP bacteraemia despite phenotypic susceptibility, in patients who were largely immunocompetent.^21^ This clinical observation suggests that the early killing seen *in vitro*—bactericidal activity of amikacin at 1 × MIC at 24 h in 60% of AME-positive isolates—may not translate into clinical benefit.

Genomic characterization helped address potential confounding by lineage. Twelve of the 13 clinical isolates, including two AME-negative isolates (S1 and S2) and all 10 AME-positive isolates, belonged to ST307 and harboured *bla*_KPC-2_, indicating that the comparison was largely made within a single clonal background. The remaining AME-negative isolate (S3) belonged to ST65 and carried *iuc1*, *iro1*, and *rmpA2*, consistent with a hypervirulent background; this may account for part of the baseline mortality difference observed under saline treatment. Nonetheless, residual confounding by lineage, virulence determinants, or other genomic differences cannot be excluded.

Conventional AST assumes that MIC thresholds determine efficacy. Our findings suggest that phenotypic susceptibility alone may not fully capture amikacin activity against CRKP harbouring *aac(6′)-Ib-cr*. However, several features of our design limit direct clinical extrapolation. We used static concentrations and single-dose administration, which do not reproduce human pharmacokinetics, where peak concentrations, AUC/MIC ratios, and post-antibiotic effects contribute to efficacy.^22–25^ The reduced efficacy observed here may therefore overestimate the effect expected under clinical dosing.

This study had several limitations. First, AME-negative isolates were uncommon in our bacteraemia collection, resulting in an imbalanced comparison (3 versus 10 isolates) and limited statistical power. Second, our study evaluated multiple strains at a single drug concentration in monotherapy and thus did not provide information on dose–response relationships. As aminoglycosides are commonly used as part of combination regimens in patients, future studies are warranted to assess combinations such as amikacin–ceftazidime–avibactam or amikacin–meropenem. Third, the time-kill experiments were performed once per isolate, which may have limited the assessment of experimental variability. Fourth, ceftazidime and avibactam concentrations were not quantified during the 72-h time-kill experiments. Although thermal degradation was likely limited,^20^ we cannot exclude incomplete inhibition of KPC-2 by avibactam, which may have contributed to the variable ceftazidime–avibactam activity.

In summary, carriage of AME gene *aac(6′)-Ib-cr* was associated with reduced amikacin efficacy in CRKP despite phenotypic susceptibility, and this association was not explained by MIC alone. These findings support a role for *aac(6′)-Ib-cr* as a treatment effect modifier rather than a binary resistance determinant. Whether genotypic characterization adds value to MIC-based interpretation warrants evaluation under clinically relevant dosing.

## Supplementary Material

dlag239_Supplementary_Data

## Supplementary data

Figure S1 and Table S1 are available as Supplementary data at *JAC-AMR* Online.

## References

[1] Logan LK, Weinstein RA. The epidemiology of carbapenem-resistant Enterobacteriaceae: the impact and evolution of a global menace. J Infect Dis 2017; 215(Suppl 1): S28–36. 10.1093/infdis/jiw282 PMC5853342 28375512

[2] Bush K, Fisher JF. Epidemiological expansion, structural studies, and clinical challenges of new β-lactamases from gram-negative bacteria. Annu Rev Microbiol 2011; 65: 455–78. 10.1146/annurev-micro-090110-102911 21740228

[3] Patel G, Bonomo R. “Stormy waters ahead”: global emergence of carbapenemases. Front Microbiol 2013; 4: 48. 10.3389/fmicb.2013.00048 PMC3596785 23504089

[4] Shields RK, Nguyen MH, Chen L et al Ceftazidime-avibactam is superior to other treatment regimens against carbapenem-resistant *Klebsiella pneumoniae* bacteremia. Antimicrob Agents Chemother 2017; 61: e00883-17. 10.1128/AAC.00883-17 PMC5527595 28559250

[5] Shields RK, Chen L, Cheng S et al Emergence of ceftazidime-avibactam resistance due to plasmid-borne *bla*_kpc-3_ mutations during treatment of carbapenem-resistant *Klebsiella pneumoniae* infections. Antimicrob Agents Chemother 2017; 61: e02097-16. 10.1128/AAC.02097-16 PMC5328542 28031201

[6] Mikhail S, Singh NB, Kebriaei R et al Evaluation of the synergy of ceftazidime-avibactam in combination with meropenem, amikacin, aztreonam, colistin, or fosfomycin against well-characterized multidrug-resistant *Klebsiella pneumoniae* and *Pseudomonas aeruginosa*. Antimicrob Agents Chemother 2019; 63: e00779-19. 10.1128/AAC.00779-19 PMC6658738 31182535

[7] Jeon M, Jang S, Mun SJ et al Thirty-day mortality of persistent methicillin-resistant *Staphylococcus aureus* bacteremia: insights from a retrospective cohort study. Yonsei Med J 2024; 65: 770–6. 10.3349/ymj.2023.0600 PMC11605044 39609092

[8] Byun J, Jung M, Chung K et al Risk factors for failure to eradicate infection after single arthroscopic debridement in septic arthritis of a native knee joint. Yonsei Med J 2025; 66: 295–301. 10.3349/ymj.2024.0190 PMC12041402 40288901

[9] Rebelo AR, Bortolaia V, Leekitcharoenphon P et al One day in Denmark: comparison of phenotypic and genotypic antimicrobial susceptibility testing in bacterial isolates from clinical settings. Front Microbiol 2022; 13: 804627. 10.3389/fmicb.2022.804627 PMC9226621 35756053

[10] Garneau-Tsodikova S, Labby KJ. Mechanisms of resistance to aminoglycoside antibiotics: overview and perspectives. MedChemComm 2016; 7: 11–27. 10.1039/C5MD00344J PMC4752126 26877861

[11] Wright GD . Aminoglycoside-modifying enzymes. Curr Opin Microbiol 1999; 2: 499–503. 10.1016/S1369-5274(99)00007-7 10508725

[12] Ramirez MS, Tolmasky ME. Aminoglycoside modifying enzymes. Drug Resist Updat 2010; 13: 151–71. 10.1016/j.drup.2010.08.003 PMC2992599 20833577

[13] Bremmer DN, Clancy CJ, Press EG et al KPC-producing *Klebsiella pneumoniae* strains that harbor AAC(6′)-Ib exhibit intermediate resistance to amikacin. Antimicrob Agents Chemother 2014; 58: 7597–600. 10.1128/AAC.03831-14 PMC4249530 25288089

[14] Butler DA, Rana AP, Krapp F et al Optimizing aminoglycoside selection for kpc-producing *Klebsiella pneumoniae* with the aminoglycoside-modifying enzyme (AME) gene *aac(6′)-Ib*. J Antimicrob Chemother 2021; 76: 671–9. 10.1093/jac/dkaa480 PMC7879147 33326561

[15] Tsai CJ, Loh JM, Proft T. *Galleria mellonella* infection models for the study of bacterial diseases and for antimicrobial drug testing. Virulence 2016; 7: 214–29. 10.1080/21505594.2015.1135289 PMC4871635 26730990

[16] Konkel ME, Tilly K. Temperature-regulated expression of bacterial virulence genes. Microbes Infect 2000; 2: 157–66. 10.1016/S1286-4579(00)00272-0 10742688

[17] Browne N, Heelan M, Kavanagh K. An analysis of the structural and functional similarities of insect hemocytes and mammalian phagocytes. Virulence 2013; 4: 597–603. 10.4161/viru.25906 PMC3906293 23921374

[18] Ignasiak K, Maxwell A. *Galleria mellonella* (greater wax moth) larvae as a model for antibiotic susceptibility testing and acute toxicity trials. BMC Res Notes 2017; 10: 428. 10.1186/s13104-017-2757-8 PMC5576310 28851426

[19] Barton TE, Duignan L, Kadioglu A et al *Galleria mellonella* as an antimicrobial screening model. J Vis Exp 2024; 212: e67210. 10.3791/67210 39465915

[20] Zhou J, Qian Y, Lang Y et al Comprehensive stability analysis of 13 β-lactams and β-lactamase inhibitors in *in vitro* media, and novel supplement dosing strategy to mitigate thermal drug degradation. Antimicrob Agents Chemother 2024; 68: e01399-23. 10.1128/aac.01399-23 PMC10916406 38329330

[21] Seong JE, Ahn SM, Han M et al Microbiological and clinical impact of aminoglycosides on KPC-producing *Klebsiella pneumoniae* bacteraemia with aminoglycoside-modifying enzymes. Diagn Microbiol Infect Dis 2026; 114: 117111. 10.1016/j.diagmicrobio.2025.117111 40961726

[22] Avent ML, Rogers BA, Cheng AC et al Current use of aminoglycosides: indications, pharmacokinetics and monitoring for toxicity. Intern Med J 2011; 41: 441–9. 10.1111/j.1445-5994.2011.02452.x 21309997

[23] Craig WA . Pharmacokinetic/pharmacodynamic parameters: rationale for antibacterial dosing of mice and men. Clin Infect Dis 1998; 26: 1–12. 10.1086/516284 9455502

[24] Drusano GL, Louie A. Optimization of aminoglycoside therapy. Antimicrob Agents Chemother 2011; 55: 2528–31. 10.1128/AAC.01314-10 PMC3101448 21402835

[25] Drusano GL, Liu W, Fikes S et al Interaction of drug- and granulocyte-mediated killing of *Pseudomonas aeruginosa* in a murine pneumonia model. J Infect Dis 2014; 210: 1319–24. 10.1093/infdis/jiu237 PMC4271070 24760199

