# Supplementary material for: Amikacin activity against carbapenem-resistant Klebsiella pneumoniae without phenotypic aminoglycoside resistance: impact of aminoglycoside-modifying enzyme (AME) gene aac(6′)-Ib-cr in vitro and in vivo
Source: JAC Antimicrob Resist. 2026 Nov 2;8(6):dlag239. doi: 10.1093/jacamr/dlag239 (PMC13724272; doi:10.1093/jacamr/dlag239)
Supplement: Supplementary file 1 [file dlag239_supplementary_data.zip › Supplementary data.docx]

**Supplementary Table 1. Change in bacterial density at 24 h (Δlog_10_ cfu/ml from the starting inoculum) and regrowth from nadir to 72 h following treatment with amikacin and ceftazidime–avibactam**

| Strain | Amikacin | | Ceftazidime–avibactam | |
| --- | --- | --- | --- | --- |
|  | 24h | Regrowth | 24h | Regrowth |
| Positive control ^a^ | -4.97 | 3.22 | -5.24 | 0.00 |
| S1 | -2.81 | 3.32 | +2.73 | 5.50 |
| S2 | -4.73 | 0.00 | -4.83 | 0.00 |
| S3 | -5.30 | 0.00 | -5.23 | 0.00 |
| R1 | -1.56 | 4.12 | -5.71 | 0.00 |
| R2 | -3.43 | 5.82 | -4.94 | 1.32 |
| R3 | -4.88 | 2.53 | -2.11 | 2.72 |
| R4 | +1.58 | 5.34 | -5.33 | 0.00 |
| R5 | -1.92 | 0.48 | -4.86 | 0.00 |
| R6 | -3.42 | 1.34 | -0.25 | 4.16 |
| R7 | -3.74 | 0.00 | -3.95 | 0.30 |
| R8 | +0.91 | 3.74 | -5.54 | 0.00 |
| R9 | -3.91 | 0.00 | -2.00 | 1.66 |
| R10 | -3.71 | 0.18 | -2.42 | 0.44 |
| AME-negative (S1–S3) | -4.28±1.30 | 0 (0–3.32) | -2.93±4.07 | 0 (0–5.5) |
| AME-positive (R1–R10) | -2.41±2.16 | 1.94 (0–5.82) | -3.71±1.89 | 0.37 (0–4.16) |
| ^a^ Positive control refers to *K. pneumoniae* NCCP 14764 included for reference comparison.  Group summaries are shown as mean ± SD (24 h killing) and, for regrowth, as median (range). | | | | |
